# Occupational risks to pregnant obstetrics and gynaecology trainees and physicians: Is it time to think about this?

**DOI:** 10.34763/jmotherandchild.20222601.d-22-00006

**Published:** 2022-07-20

**Authors:** Mishu Mangla

**Affiliations:** Department of Obstetrics & Gynaecology, All India Institute of Medical Sciences, Bibinagar, Hyderabad, India

**Keywords:** Occupational risks, pregnancy, health care workers, obstetrician, gynaecologist, teratogenesis, radiation exposure, violence against doctors

## Abstract

The proportion of women in the workforce has been steadily increasing worldwide. Women now constitute approximately 75% of the global health workforce and almost 90% in nursing and midwifery professions. The present times have witnessed a dramatic gender shift in the speciality of obstetrics and gynaecology. Women now comprise a significant proportion of practicing obstetrics and gynaecology specialists all over the world. In 2018, more than 80% of resident doctors and nearly 60% of physicians in the speciality were female, far exceeding any other surgical speciality. Most resident doctors and a significant proportion of practising physicians in obstetrics and gynaecology are in the reproductive age group. They will become pregnant at some point in their training program or career. The present review focuses on all work-related exposure risks for pregnant obstetrics and gynaecology professionals. It discusses the risks of infectious diseases, radiation, stress, violence against doctors, and even peer support (or lack of it) that can have deleterious effects on the health of pregnant physicians and the health of their unborn foetuses.

## Introduction

The proportion of women in the workforce has been steadily increasing worldwide. Women now constitute approximately 75% of the global health care workforce [1] and almost 90% in nursing and midwifery professions [2]. In India, females form 38% of all health care workers (HCW) and about 16.8% of allopathic doctors. Roughly 1 in every 3 HCW is a female [3] . The present times have witnessed a dramatic gender shift in the speciality of obstetrics and gynaecology. Women, now comprise a significant proportion of practicing obstetrics and gynaecology specialists all over the world [4]. In 2018, more than 80% of resident doctors and nearly 60% of physicians in the speciality were female, far exceeding any other surgical speciality [5]. This is in stark contrast to 2012, when women comprised >50% of Fellows and Junior Fellows in the American College of Obstetricians and Gynecologists [5]. In India, this figure is much more than 90%, as the patients in this world favour female physicians for their gynaecological issues. The male trainees often feel bias because of patients preferring female physicians [6].

The majority of resident doctors and a significant proportion of physicians in Obstetrics and Gynaecology are in the reproductive age group. They are or will become pregnant at some point in their training program or career. Pregnant HCWs are faced with numerous challenges, as they need to balance their health and the health of their unborn child along with that of their patients. Proper performance of their duties may at times constitute a risk to their own health. Although pregnant women are not more susceptible to most diseases than their non-pregnant counterparts, the consequences of even a mild infection can be far-reaching. Rubella and chickenpox, although most cases are self-resolving, can lead to abortions and congenital abnormalities in the offspring. It is quite common for a health care worker to feel torn between duties towards her patients and co-workers and her responsibilities towards her family and her unborn foetus. A pregnant trainee or even a consultant physician in obstetrics and gynaecology faces unique occupational challenges and hazards. Apart from the physically taxing nature of work in the labour room, where each normal delivery needs continuous monitoring and vigilance for at least 6–8 hours, a number of other occupational risks are unique to the speciality, which probably at the time of pregnancy become matters of concern. To the best of my knowledge, there is no comprehensive review that identifies occupational risks to pregnant obstetricians and gynaecologists. This review focuses on all work-related exposure risks, such as risks of infectious diseases, radiation, stress, violence against doctors, and even peer support, or lack of support, that can have deleterious effects on the health of pregnant physicians and the health of their unborn foetuses.

## Material and methods

The recent literature related to pregnant health care workers and occupational risks was searched, from various governmental agencies including the World Health Organisation (WHO), Centers for Disease Control and Prevention (CDC), Scientific Advisory Group for Emergencies (SAGE), Occupational Safety and Health Administration (OSHA), and English peer-reviewed journals from databases such as PubMed, Scopus, Google Scholar, EMBASE, and others. The literature regarding workplace regulations for pregnant or lactating health care staff was also reviewed. The search terms used were: ‘pregnant health care worker’ AND ‘occupational risks’; OR ‘radiation exposure’; OR ‘violence against doctors’; OR ‘infectious diseases’ OR ‘physician burn out or stress; Or ‘anaesthetic gases’. The articles referring to obstetrics and gynaecology trainees and physicians were studied in detail to work out all the occupational risks faced by them during pregnancy.

### Radiation exposure

Radiation exposure in early pregnancy is very well known to be associated with teratogenic effects. Although the risk is usually overestimated, the consequences can be significant in cumulative doses. Threshold radiation effects (deterministic effects) occur over a dose threshold and result in cellular injury. Stochastic effects of radiation are incremental, appearing in a dose-response function without a threshold, and are thought to be the primary mechanism of increased risk of cancers [7]. Various agencies have given thresholds that should be taken care of once pregnancy is confirmed, to minimize the effects on the foetus, mainly during organogenesis. The International Commission on Radiological Protection recommends that after a worker declares her pregnancy, the occupational radiation dose should not exceed one mSv during the remainder of the pregnancy. The National Council on Radiation Protection and Measurements, in the United States, recommends a radiation dose limit of 0.5 mSv per month once pregnancy is confirmed to ensure a low exposure during susceptible periods of gestation [8]. The US Environmental Protection Agency recommends a limit of 5 mSv for the entire gestational period [8].

Although only very few procedures in obstetrics and gynaecology use ionizing radiation, hysterosalpingography (HSG) is a standard procedure used to evaluate the patency of fallopian tubes in women presenting with subfertility. The HSG procedure requires the radiologist or preferably a gynaecologist (who is not trained to handle ionizing radiation) to hold the cannula and inject the contrast medium into the patient's cervix while she is being irradiated. The supporting personnel also remain close to the patient. Though a lead apron is worn, frequent or multiple procedures can lead to significant exposure to ionizing radiation, which can be worrisome, especially in the first trimester due to teratogenic effects [8]. A few studies have demonstrated that the dose to the extremities may also be significant enough to warrant monitoring, especially when the procedure is done frequently.

Sentinel lymph node mapping, used to identify the affected or sentinel node in gynaecological malignancies, also uses radioactive tracers such as Technetium-99 to identify the affected nodes. Although preliminary studies have indicated that exposure usually falls within the safe limits, many factors, such as time from injection to surgery and distance between the patient's injection site and the surgeon's abdomen, play a significant role [9]. Physicians and trainees predominantly dealing with gynaecological malignancy surgeries, should consider the cumulative dose received. Due to the fear of discrimination by their peers or senior consultants, some of the trainees or resident doctors prefer not to disclose their pregnant status till late. If unaware of the risks during such procedures, they may expose themselves to ionizing radiation, causing inadvertent self-harm. There is a need for a cordial work environment in which the resident doctors can declare their pregnant status, free from the fear of discrimination, and can hold posts in areas that do not involve radiation exposure.

### Infectious diseases

Women during pregnancy are more susceptible to certain viral infections, predominantly due to impaired pathogen clearance and hormonal and immunological alterations. The risk for health care workers, including doctors, is increased manifold mainly due to the prolonged contact with infected patients and that too in closed areas such as birthing suites, high dependency units, and intensive care units. If a pregnant health care worker acquires viral infections such as rubella, cytomegalovirus, herpes simplex, and varicella, particularly at the time of organogenesis, it can be devastating for the foetus. The teratogenic effects of these viruses are well known. Pregnant doctors should not be involved in the care of patients with these infectious diseases.

The current pandemic of COVID-19 has made this risk of infectious diseases even more apparent. CDC recognizes health care workers, including doctors, nurses, dentists, paramedics, emergency medical technicians, laboratory personnel collecting and handling samples from infected persons, and morgue workers performing autopsies as the group at highest risk of acquiring Coronavirus [10]. Among health care professionals, certain professionals such as anaesthesiologists, otorhinolaryngologists, dentists, ophthalmologists are at exceptionally high risk because their work demands proximity with patients’ respiratory tracts. Obstetricians also come under very high risk, mainly due to prolonged exposure, especially during labour. CDC and WHO recommend using N95 masks for health care workers, especially during the care of patients with diseases that involve droplet transmissions, such as tuberculosis, severe acute respiratory syndrome (SARS), and COVID-19 [11]. Even after complying with proper protection and preventive measures and using personal protective equipment to protect themselves, health care workers have been affected by the disease.

During the initial months of the pandemic, a few efforts were taken to limit the amount of exposure, such as delaying elective surgical procedures and undertaking only emergency ones. However, these practice guidelines cannot be extended to specialities like obstetrics. Every case presenting in labour or needing labour induction due to feto-maternal indications can be treated as an emergency, as any delay can be life-threatening. Obstetrics is probably the only speciality in medicine where the number of cases and surgeries did not decrease, despite the fear of infection. The risks extend beyond the pandemic period and apply to other infectious diseases such as influenza and tuberculosis.

### The dilemma with personal protective equipment in pregnancy

Pregnancy is associated with profound changes in normal respiratory physiology. Dyspnoea is usually a common symptom in late pregnancy. Both mechanical factors (due to the enlarged gravid uterus) and hormonal factors play a role in this. Oxygen consumption increases from the first trimester, increasing by around 30% per term due to maternal metabolic processes and foetal demands [12]. Increased oestrogen causes hyperaemia, oedema, hypersecretion, and friability of the mucosa of the respiratory tract [13]. There is an increase in the number and sensitivity of hypothalamic and medullary progesterone receptors in pregnancy, leading to a rise in the sensitivity of peripheral chemoreceptors to hypoxic conditions [14]. Progesterone also leads to a decreased threshold and increased respiratory centre sensitivity to carbon dioxide. These physiological changes increase the load on the respiratory system in pregnancy.

Keeping in mind the airborne transmission of COVID-19 [15], the Scientific Advisory Group for Emergencies (SAGE) [16] even recommends that HCW caring for patients with suspected or confirmed COVID-19 may need higher grade protective masks, such as FFP3 masks equivalent to N99, to protect them from contracting the virus through the air. Although a few studies suggest that these masks are not associated with adverse effects in pregnancy, these studies are primarily restricted by limited time of exposure, i.e., a maximum of one hour [15]. But in today's scenario, the duration of mask-wearing by pregnant women, especially health care workers, is at least 6–8 hours at a stretch. Several side effects have been reported in health care workers using these face masks for a prolonged duration. These include headache, dryness in the eyes and nose, acne, epistaxis, skin breakdown, and even impaired cognition [17]. Pregnant women, especially in the late second or third trimester, may not be able to maintain their required minute ventilation while breathing through N95 respirators. The workload on breathing increases significantly, leading to decreased oxygen uptake and increased carbon dioxide concentration [18]. Hypoxia and hypercarbia, mainly due to re-breathing caused by retained carbon dioxide in the mask's dead space, occur on prolonged mask usage [19] These changes are evident even at rest and may be exacerbated on mild to moderate exertion. Long-term exposure of the foetus to this increased carbon dioxide level has not been studied. However, some studies suggest that it affects foetal cerebral oxygenation, which may be by regulating the cerebral blood flow and shifting the oxyhaemoglobin dissociation curve [19].

Pregnant women with respiratory ailments such as bronchial asthma or other chronic lung diseases could be at much higher risk. The use of medical and surgical masks and other external airflow resistive load devices has been found to impact some hemodynamic parameters such as diastolic blood pressure and mean blood pressure [19] significantly, in pregnant women and non-pregnant women alike. Although the effect noted was mild, even an increase of 10 mm Hg in a patient with preeclampsia or chronic hypertension could be harmful to the mother and the unborn foetus.

### Sharps injuries and bloodborne infections

All surgeons have a very high risk of needle-stick injury, and obstetrics and gynaecology as a speciality are no different. Resident doctors are at an exceptionally high risk as they are not trained in personal protection measures, and most of them are learning to hold and manipulate the instruments for the first time. A survey of around 700 resident doctors found that almost 99% of them had experienced a sharps injury [20]. The probability of acquiring infection from large-bore needle-stick injury has been reported to be as high as 40% in workers not vaccinated against hepatitis B virus, 1.8% for hepatitis C virus, and 0.3% for human immunodeficiency virus (HIV) [21].

Until the time they are properly trained in handling and manipulating surgical instruments and needles, pregnant resident doctors should not be involved in the surgery of patients with HIV, hepatitis B, and hepatitis C. Further adequate vaccination and good antibody titers against hepatitis B should be a rule for trainee doctors joining any surgical speciality. They should also be trained to handle blood and body fluid spills and be adequately informed regarding post-exposure prophylaxis in case of accidental needle-stick injury.

### Physician burnout and stress

Pregnancy during residency and speciality training in medicine and surgery is challenging. The residency period, especially in clinical specialties like obstetrics and gynaecology, is marked by long duty hours, rotating night shifts, and prolonged standing. Working long hours during the first trimester of pregnancy is associated with threatened abortion and preterm birth [22]. A recent survey on 347 general surgeons, who had at least one pregnancy during residency, reported unmitigated work schedules during pregnancy. There is a negative stigma associated with pregnancy during training. They were also dissatisfied with maternity leave options and inadequate lactation and childcare support. They also desired a better mentorship on work-life integration [23].

Inadequate support by the co-doctors is expected because they are themselves engrossed in their heavy duties. Several studies in the past have stressed that most residents felt inconvenienced by the presence of pregnant or lactating colleagues, as they were forced to cover their responsibilities during their absence (24). Resident doctors during pregnancy and lactation face unique challenges such as arranging for child care during their extended period of absence, maintaining lactation during intense night duties, and frequent breaks for pumping breast milk to ensure proper milk output. Inadequate policies related to pregnancy and parenting may sometimes even adversely affect their career preferences, sometimes even promoting them to quit their career as medical professional [25].

### Peer support

Fulfilling lactation and child care goals is another challenge for health care workers across all specialities. Maintaining an adequate breast milk supply requires either frequent feeding or frequent pumping, both of which need frequent short breaks in the working schedule. More than half of the doctors and supporting staff opt to quit breastfeeding at an earlier stage than they wished. The nature of the work of health care professionals is such that taking even a short break without proper replacement can even cost lives. There is presently no provision adjustment in the nature of duties of pregnant and lactating health care workers. A written policy regarding avoidance of long duty hours and prolonged standing, and provision of intermittent periods of rest, should be made and brought into practice in health care settings. Provision of lactation rooms with facilities for pumping and storing breast milk should be mandatory. Lactating employees should be provided with frequent short breaks to pump or breastfeed. Although some hospitals do offer an in-house creche and child care facility, taking the baby to hospitals is again a dilemma, especially at the time of the spread of a pandemic which is highly infectious.

### Exposure to anaesthetic gases and surgical smoke and other chemicals

Nitrous oxide and halogenated agents constitute the predominant inhalational agents used for anaesthesia in operation theatres. When inhalational agents are used for induction predominantly for day care procedures or minor surgeries in gynaecology, some waste gases are inadvertently released into the operating room and inhaled by surgeons and their supporting staff. These gases have been associated with adverse pregnancy outcomes such as spontaneous abortions and congenital anomalies in the foetus when inhaled by pregnant women, especially during earlier gestation [26]. Therefore, adequate scavenging systems should be a must in all operation theatres to minimize exposure [27].

*Surgical smoke* refers to waste gases emitted in the operation theatres due to the burning of tissues with energy sources such as electrocautery. The content of surgical smoke includes water gases containing chemicals such as benzene, 1,2-dichloroethane, and toluene, which are associated with miscarriages, congenital birth defects, foetal growth restriction [28], and preterm labour [29]. Many studies have found a very high concentration of fine and ultrafine particulate matter when smoke was released during laparoscopic procedures. Although these particles and chemicals have not been studied in much detail, the effects that these particles and chemicals have on the unborn foetus could be significantly grave. Cytoreductive surgery and hyperthermic intraperitoneal chemotherapy (HIPEC) are increasingly used as a treatment modality for ovarian malignancies and peritoneal carcinomatosis. The chemotherapy agents used include mitomycin c and platinum-based compounds such as cisplatin and carboplatin. Pregnant doctors can be exposed through both inhalation and skin contact. These agents are associated with multiple harmful effects in pregnancy, including miscarriage and congenital malformations [30]. Current recommendations are that pregnant women or those planning to become pregnant should keep themselves away from chemotherapy agents and in operation theatres where HIPEC is being done [31].

### Violence against doctors

It is a paradox that a profession as noble as health care, with the mission to care for people when their need for care is at a maximum, that is, when they are unwell or terminally ill, is at significant risk of workplace violence. The World Health Organisation defines workplace violence as 'incidents where staff is abused, threatened or assaulted in the circumstances related to their work, including commuting to and from work, involving an explicit or implicit challenge to their safety, well-being or health' [32]. It has been observed that around one-fourth of all violent accidents at work occur in the health care sector, and more than half of all health care workers have experienced violence in some form at their workplace [33]. Women, predominantly of the reproductive age group, represent nearly 80% of the health care workforce [34]. The effects of direct physical violence are well known in foetal injury and death, abruptio placentae, and premature rupture of membranes. The indirect effects of verbal, physical, and even sexual abuse include psychological stress and anxiety, which is well known to cause adverse pregnancy outcomes [35].

### What can be done?

Pregnant health care professionals including those specialising in obstetrics and gynaecology are themselves often not prepared to identify risk factors that can adversely affect their health at the work place. Occupational risk assessment models which incorporate all possible risk factors [36], should be implemented in all hospitals. Flexible working policies for their pregnant employees, including avoidance of night shifts and long shifts, especially during the trimesters that involve the highest risk to the foetus have been rightly introduced by some universities such as Indiana University's emergency and internal medicine programs. Employment conditions that are pregnancy- and breastfeeding-friendly are the need of the hour.

## Conclusion

The major employment issues faced by pregnant health care workers include pregnancy-related discrimination, accommodations in the distribution of work or duties, keeping in mind the health of mother-foetus duo, job-protected leave, and wage replacement while on maternity leave. Employment conditions that create more optimal work environments for pregnant employees are the need of the hour.

## Key points

- Women, predominantly of the reproductive age group, constitute a significant proportion of the health care work force.
- Pregnant obstetrics and gynaecology trainees and physicians face numerous occupational risks, including those of infectious diseases, radiation exposure, stress and burnout, violence against doctors, and even lack of peer support.
- Employment conditions that create more optimal work environments for pregnant employees are the need of the hour.
